# Hospital Meetings, &c.

**Published:** 1897-03-20

**Authors:** 


### HOSPITAL MEETINGS, &C.

### ROYAL HOSPITAL FOR DISEASES OF THE CHEST.

The Lord Mayor presided over the annual court of governors of the Royal Hospital for Diseases of the Chest, which was held at the hospital on Monday, 15th inst. During the year 1896 742 in-patients and 6,583 new out-patients were treated, a slight increase on the previous year. The ordinary income amounted to £7,305 against an ordinary expenditure of £7,460. In addition legacies were received amounting to £2,803. Annual subscriptions increased from £2,218 to £2,252, and as a proof of the difficulty experienced in increasing this source of income the council pointed out that, although the new subscriptions obtained during the year amounted to £185, the actual increase in the list was only £34. The Lord Mayor, in moving the adoption of the report, said he had seldom seen anything which appealed to him more than the Royal Chest Hospital, which recognised no political creed, no religious barrier, and no difference in sex or age. Mr. T. Andros de la Rue (chairman of the Council of Management) seconded, and the report was adopted. Votes of thanks to the various officials and the re-election of the retiring members of the council concluded the business of the meeting.

### HOME HOSPITALS' ASSOCIATION.

The nineteenth annual meeting of the Home Hospitals' Association for paying patients was held at Fitzroy House, Fitzroy Square, W., on Tuesday, the 16th inst., Mr. Hugh Hammersley in the chair. The Chairman moved, and Dr. Hare seconded, the adoption of the report, which was carried. The report stated that the past year had been one of satisfactory progress. Fitzroy House, unlike all the other homes, is not conducted on commercial principles; nor, on the other hand, is there anything of an eleemosynary character about it. It was established to provide accommodation for those who can afford to pay a reasonable sum for their lodging, nursing, and diet when ill; the object was not that of gain, but the advantage of the community and the relief of those who are inadmissible at the general hospitals, and who yet have not the means to provide, when ill, the necessary comforts at their own homes. How well it has answered its purpose this and the previous reports amply testify. During 1896 317 applications were received for admission; of this number 283 were admitted, viz., 139 male patients and 144 female patients; 47 friends of patients were also received into the home attached to the hospital. Sixty-seven medical practitioners have had patients at the hospital in 1896, as against 66 in 1895. The election of the committee and votes of thanks to the Medical Board and to the chairman concluded the meeting.

